# Characterization of an Estrogen Receptor α-Selective ^18^ F-Estradiol PET Tracer

**DOI:** 10.1055/s-0044-1786518

**Published:** 2024-06-18

**Authors:** Pavel Sluka, Uwe Ackermann, Angela Rigopoulos, Hady Wardan, Carmel Pezaro, Ingrid J.G. Burvenich, Andrew M. Scott, Ian D. Davis

**Affiliations:** 1 Eastern Health Clinical School, Monash University, Box Hill, VIC, Australia; 2 Department of Molecular Imaging and Therapy, Austin Hospital, Heidelberg, VIC, Australia; 3 Olivia Newton-John Cancer Research Institute, Heidelberg, VIC, Australia; 4 School of Cancer Medicine, La Trobe University, Bundoora, VIC, Australia; 5 The University of Melbourne, Parkville, VIC, Australia; 6 Department of Oncology, Eastern Health, Box Hill, VIC, Australia

**Keywords:** estrogen receptor α, estrogen receptor β, positron emission tomography, radiopharmaceuticals, competitive binding

## Abstract

**Objective** Conventional imaging of cancer with modalities such as computed tomography or magnetic resonance imaging provides little information about the underlying biology of the cancer and consequently little guidance for systemic treatment choices. Accurate identification of aggressive cancers or those that are likely to respond to specific treatment regimens would allow more precisely tailored treatments to be used. The expression of the estrogen receptor α subunit is associated with a more aggressive phenotype, with a greater propensity to metastasize. We aimed to characterize the binding properties of an ^18^ F-estradiol positron emission tomography (PET) tracer in its ability to bind to the α and β forms of estrogen receptors in vitro and confirmed its binding to estrogen receptor α in vivo.

**Methods** The ^18^ F-estradiol PET tracer was synthesized and its quality confirmed by high-performance liquid chromatography. Binding of the tracer was assessed in vitro by saturation and competitive binding studies to HEK293T cells transfected with estrogen receptor α ( *ESR1* ) and/or estrogen receptor β ( *ESR2* ). Binding of the tracer to estrogen receptor α in vivo was assessed by imaging of uptake of the tracer into MCF7 xenografts in BALB/c nu/nu mice.

**Results** The ^18^ F-estradiol PET tracer bound with high affinity (94 nM) to estrogen receptor α, with negligible binding to estrogen receptor β. Uptake of the tracer was observed in MCF7 xenografts, which almost exclusively express estrogen receptor α.

**Conclusion** ^18^ F-estradiol PET tracer binds in vitro with high specificity to the estrogen receptor α isoform, with minimal binding to estrogen receptor β. This may help distinguish human cancers with biological dependence on estrogen receptor subtypes.

## Introduction

Most breast cancers express estrogen receptors (ERs), which indicate better prognosis and predict responsiveness to hormone therapy. 1 2 3 Conventional immunohistochemical methods of determining ER status of breast cancers are limited by sampling error and tumor heterogeneity. 4

Development of positron emission tomography (PET) imaging for ERs with the 16α-[ ^18^ F]fluoro-17β-estradiol ([ ^18^ F]FES) radiotracer has permitted in vivo evaluation of ER expression. 5 6 As with immunohistochemical staining, [ ^18^ F]FES is able to detect breast cancers that express ER 7 8 9 and that can respond to hormone therapy. 10 [ ^18^ F]FES uptake correlates with ER immunohistochemistry (IHC) in breast cancer 8 9 11 12 and can be combined with[ ^18^ F]-fluorodeoxyglucose ([ ^18^ F]FDG) to identify heterogeneity of a patient's disease and potentially identify lesions that are functionally ER-negative ([ ^18^ F]FES ^–^ /[ ^18^ F]FDG ^+^ ) despite some lesions expressing ER by IHC. 13 14 [ ^18^ F]FES is also able to interrogate the ER status of lesions that are not amenable to biopsy either due to their location or number. 15 This is particularly useful in patients with recurrent or metastatic disease. 16

In treatment-naïve patients, [ ^18^ F]FES uptake at baseline had a positive predictive value of 79 to 87% and a negative predictive value of 88 to 100% for response to hormone therapy, 10 17 higher than that reported for IHC. [ ^18^ F]FES also allows for early success of therapy to be determined. A reduction in [ ^18^ F]FES uptake at day 28 of treatment when compared to day 1 correlates with increased progression-free survival. 18

Although the primary reported metric for ER immunohistochemical status is positive or negative, the biology is complicated by the presence of two ER subtypes: ERα and ERβ. ERα expression and function are linked to a more aggressive phenotype, 19 whereas ERβ inhibits migration and proliferation, 20 and expression is linked to less aggressive phenotypes with improved survival. 21 In support of this, loss of ERβ is associated with a more aggressive cancer phenotype. 22 A similar association of ERα-“progressive” and ERβ-“suppressive” phenotypes is also seen in other cancers such as prostate cancer. 23

The ability to identify both biologically aggressive and nonaggressive cancers would be highly useful, and would provide the treating team valuable prognostic and predictive data. Anecdotal reports indicate that [ ^18^ F]FES binds with higher affinity to ERα than ERβ. In endometrial cancer, [ ^18^ F]FES uptake correlated with immunohistochemical expression of ERα but not ERβ, 24 while mice with ERα-knockdown tumors showed lower uptake of [ ^18^ F]FES than their ERα-transfected counterparts. 25 To date, no study has investigated the clinical significance of selective imaging for ER isoforms. The aim of our study was to determine the affinity of [ ^18^ F]FES for the individual ER subtypes using cell lines that contained only either ERα or ERβ with a view to guiding interpretation of [ ^18^ F]FES PET.

## Materials and Methods

### Generation of the ^18^ F-Estradiol PET Tracer

The cyclic sulfate 3-O-methoxy-methyl-16β,17β-epiestriol-O-cyclic sulfone was used as precursor for the synthesis of [ ^18^ F]FES. 26 The production of [ ^18^ F]FES was fully automated using the FlexLab module. No-carrier-added [ ^18^ F]fluoride was produced by the ^18^ O(p, n) ^18^ F nuclear reaction in a niobium target using the IBA Cyclone 18/9 using [ ^18^ O]H _2_ O, at Austin Health, Centre for PET. Typical irradiation parameters were 40 µAh for 45 min, which produced 1.5 Ci of ^18^ F. After transfer from the target, ^18^ F was trapped on a quaternary methyl ammonium cartridge and eluted using a solution containing 3.45 mg of anhydrous K _2_ CO _3_ (0.025 mmol) and 20 mg of Kryptofix 2.2.2 (0.053 mmol) in 0.4 mL of acetonitrile plus 0.2 mL of water. Azeotropic evaporation to dryness with 1 mL of dry acetonitrile gave the anhydrous potassium [ ^18^ F]fluoride complex used in the labelling experiments.

The radiolabeling precursor (2 mg) was dissolved in acetonitrile (1 mL) and added to the dried potassium [ ^18^ F]fluoride complex. The mixture was heated to 110°C for 8 minutes, followed by cooling to 90°C and evaporation to dryness. A solution of 44 mM H _2_ SO _4_ in ethanol (3 mL) was added and the mixture heated to 110°C to afford [ ^18^ F]FES. The radiotracer was prepurified by adding 6 mL of water and trapping on a C-18 cartridge. [ ^18^ F]FES was eluted using 1.5 mL of acetonitrile and diluted with 3.5 mL of ammonium formate (0.1M) for purification by high-performance liquid chromatography (HPLC). Semipreparative HPLC separation of [ ^18^ F]FES from by-products was achieved using a Phenomenex Gemini column (250 × 10mm). Acetonitrile (A) and 0.1 M ammonium formate (B) were used as the mobile phase at a flow rate of 4 mL/min and a gradient elution technique was used for purification: 0 to 20 min: 35 to 55% A. Retention time of [ ^18^ F]FES was 12 minutes. The fraction containing the radiotracer was collected and reformulated in 10 mL of 10% ethanol in saline using the solid phase extraction technique. 27

A Shimadzu HPLC system equipped with a 20μL injection loop, an SPD-20A ultraviolet visual detector, and two LC-20AD solvent pumps for high pressure mixing of mobile phase were used for quality control. The Bioscan FC-4000 dual BGO PET coincidence detector was used for the detection of radioactive compounds. The stationary phase was a Phenomenex Gemini NX C-18, 5µm RP column, 150 × 4.6 mm. Acetonitrile (A) and water (B) with 0.1% formic acid were used as the mobile phase at a flow rate of 0.5 mL/min and a gradient elution technique was used for analysis: 0 to 18 minutes: 5–90% A, 18–30 minutes: isocratic 90% A. Specific radioactivity was measured using a mass standard curve of known concentrations of [ ^18^ F]FES.

### Cell Lines

HEK293T and MCF7 cells were obtained from the ATCC and maintained in RPMI-1640 medium containing 1× GlutaMAX (Gibco, VIC, Australia) supplemented with 10% fetal bovine serum (Gibco, VIC, Australia) and 100U/mL penicillin plus 100 µg/mL streptomycin (Gibco, VIC, Australia).

### Estrogen Receptor Plasmid Constructs

The ER expression plasmids used in this study were a pcDNA3.1 expression vector containing full-length human *ESR1* (ERα) 28 and a pCMV5 expression vector containing full-length human *ESR2* (ERβ). 29

### Transient Transfection of HEK293T Cells with ER Subtypes

#### Transfection

HEK293T cells were transiently transfected with either *ESR1* ( *ERα* ) alone (1µg plasma DNA), *ESR2* ( *ER* β) alone (1µg plasma DNA), or both *ESR1* (0.5µg plasma DNA) and *ESR2* (0.5µg plasma DNA). Transfection constructs were graciously gifted by Chu et al. 28 Control transfections were conducted without DNA. For transfection, 3.8 × 10 ^5^ HEK293T cells were seeded into 12-well plates (∼50% confluency) and cultured for 6 hours at 37°C/5% CO _2_ to allow plating, after which 100 µL of a transfection reagent cocktail containing 1 µg total DNA, 2µL P3000 Reagent (Invitrogen), and 3µL lipofectamine 3000 Reagent (Invitrogen) in OptiMEM medium (Life Technologies) were added directly to each well. Cells were cultured for a further 40 hours at 37°C before performing binding analysis. The 40-hour period of culture following transfection was empirically identified as producing highest levels of transfected mRNA and protein (data not shown).

#### Validation of Transfection by Real-Time RT-PCR

Success of transfection at the RNA level was determined by real-time reverse-transcription polymerase chain reaction (RT-PCR) for ERα and ERβ subtypes. For these experiments, HEK293T cells were transfected as above. Total RNA was extracted using Qiagen RNeasy Mini Kits (Qiagen, VIC, Australia) according to the manufacturer's instructions with the following optional parameters: β-mercaptoethanol (Sigma-Aldrich, VIC, Australia) was added to buffer RLT, on-column DNase digestion was performed using the RNase-Free DNase Set (Qiagen, VIC, Australia), and elution was in 30µl RNase-free water. Total RNA yield and quality were assessed using a NanoDrop Lite Spectrophotometer (Thermo Fisher Scientific, VIC, Australia) and 1.5% agarose (Astral Scientific, NSW, Australia) gel electrophoresis (data not shown).

Total RNA (500ng) was reverse transcribed using the SuperScript III First-Strand Synthesis SuperMix (Invitrogen, VIC, Australia) according to the manufacturer's instructions in a 20µL volume using 2.5µM oligo(dT) and 2.5ng/µL random hexamer primers. “No RT” controls were used to confirm the absence of genomic DNA contamination.

Real-time PCR was performed using SYBR Select Master Mix for CFX (Applied Biosystems, VIC, Australia) in 10 µL reaction volumes containing 300nM forward and reverse PCR primers (Sigma-Aldrich, VIC, Australia; see Table 1 for primer sequences). For ERα and ERβ, the equivalent of 0.16µL RT reaction was used in each reaction; for the 18S rRNA housekeeper gene, the equivalent of 0.008µL RT reaction was used in each reaction. PCR cycling was performed using a CFX Connect Real-Time System (Bio-Rad, NSW, Australia) with CFX Manager v2.1 software (Bio-Rad, NSW, Australia). PCR products were analyzed by electrophoresis on 2% agarose gels.

**Table 1:** PCR primer sequences

Gene	Primer sequence	Product size (bp)	Transcript Ensembl ID
*ESR1* (ERα)	Fwd: 5′-ATC CAC CTG ATG GCC AAG-3′ Rev: 5′-GCT CCA TGC CTT TGT TAC TCA-3′	112	ENST00000406599
*ESR2* (ERβ)	Fwd: 5′-CAG CTG GGC CAA GAA GAT T-3′ Rev: 5′-CAC ATC AGC CCC ATC ATT AAC-3′	105	ENST00000341099
GREB1	Fwd: 5′-CAG GCT TTT GCA CCG AAT CT-3′ Rev: 5′-CAA AGC GTG TCG TCT TCA GCT-3′	102	ENST00000381486.7
18S rRNA	Fwd: 5′-TGG CTC ATT AAA TCA GTT ATG GTT C-3′ Rev: 5′-CTT CGG CAT GCA TTA GCT CT-3′	91	ENST00000363132

#### Validation of Transfection by Western Blotting

Western blotting was used to confirm ERα and ERβ transfection resulted in protein expression. After 40 hours of transfection, cells were washed twice with phosphate buffered saline (PBS) and lysed in the culture dish with RIPA buffer containing cOmplete Mini (Roche, VIC, Australia) protease and PhosSTOP (Roche, VIC, Australia) phosphatase inhibitor cocktails, prepared according to the manufacturer's instructions. Lysed samples were collected and incubated at 4°C for 15 minutes with head-over-tail rotation, centrifuged at 16,000 *g* for 10 minutes at 4°C, and the supernatant collected. Protein concentration was determined using the BCA Protein Assay (Pierce, VIC, Australia).

For Western blotting, 20µg protein was electrophoresed using NuPAGE Novex 4-12% Bis-Tris Mini Gels under reducing conditions using MES SDS Running Buffer (Invitrogen, VIC, Australia). A SeeBlue Pre-stained molecular weight standard (Invitrogen, VIC, Australia) was included. Protein bands were transferred to membranes using an iBlot Western Blotting System (Invitrogen, VIC, Australia) and iBlot Nitrocellulose Transfer Stacks (Invitrogen, VIC, Australia) at 20V for 7 mins.

Primary antibodies used for Western blotting were rabbit monoclonal immunoglobulin G (IgG; clone D8H8) anti-ERα (Cell Signaling Technology, MA, USA) at 1:1,000, rabbit polyclonal IgG anti-ERβ (Invitrogen, VIC, Australia) at 1:125, and mouse monoclonal IgG1 (clone AC-15) anti-β-actin (Sigma-Aldrich, VIC, Australia) at 1:8,000. Secondary antibodies were goat anti-rabbit IRDye 800CW IgG (H + L) (Li-Cor, NE, USA) at 1:15,000 for the detection of ER subtypes, and goat anti-mouse IRDye 680RD IgG (H + L) (Li-Cor) at 1:15,000 for the detection of β-actin.

All Western blotting incubations were performed at room temperature with gentle agitation unless stated otherwise. Membranes were rinsed with PBS for 5 minutes, incubated in Odyssey Blocking Buffer (Li-Cor, NE, USA) for 1 hour, and in Odyssey Blocking Buffer containing primary antibody and 0.1% Tween 20 (Sigma-Aldrich, VIC, Australia) overnight at 4°C. Each blot was probed simultaneously for β-actin and one ER subtype. Membranes were then washed with PBS containing 0.1% Tween 20 for 4 × 10 minutes and incubated in Odyssey Blocking Buffer containing secondary antibody, 0.1% Tween 20, and 0.01% SDS (Sigma-Aldrich, VIC, Australia) for 1 hour in the dark. Membranes were again washed with PBS containing 0.1% Tween 20 for 4 × 10 minutes, rinsed 2× with PBS, and scanned using an Odyssey CLx with Image Studio v3.1.4 software (Li-Cor, NE, USA).

### In Vitro Characterization of ^18^ F-Estradiol Binding

#### Binding Assays

Binding assays were conducted using transiently-transfected HEK293T cells cultured in 12-well plates, transfected as described above. Cells were washed 2× with 0.5mL binding buffer (RPMI-1640 containing 0.1% BSA (Sigma-Aldrich, VIC, Australia) and 10mM HEPES), after which hot and cold ligand were added in a final volume of 0.5mL. Cells were incubated for 2 hours at 37°C to allow binding, washed 2× with cold PBS to remove unbound ligand, lysed with 0.5mL PBS containing 0.1% Triton X-100, and transferred to fraction collector tubes. Radioactivity was measured for 30s using a Perkin Elmer Wallac Wizard 1470 Gamma Counter.

For competitive binding assays, 20,000cpm were added to each well with cold ligand titrated from 1µM to 0.98nM in doubling dilutions. For saturation binding assays, hot ligand was titrated from 10 ^5^ cpm to 100cpm in doubling dilutions. Scatchard analysis was performed by calculating the bound:free ratio and plotting against the amount of tracer bound. A linear regression line was calculated to fit the data. The slope of the regression line was used to calculate the affinity dissociation constant of the ligand.

Given the lack of binding observed with [ ^18^ F]FES to ERβ, the biological activity of transfected ERβ was confirmed. HEK293 cells transfected with ERβ alone were treated with 1uM E2 for 24 hours, cDNA was prepared and assayed by real-time PCR for GREB1, an ERβ-responsive gene. 30 These experiments were conducted as described above.

### In Vivo Characterization of ^18^ F-Estradiol Binding

MCF7 cells (10 ^6^ ), which mainly express ERα, were resuspended in 100µL Matrigel (Life Technologies, VIC, Australia) and injected subcutaneously above the right shoulder into female BALB/c nu/nu mice (n = 3; Australian Research Centre, WA, Australia). All animal studies were approved by the Austin Hospital Animal Ethics Committee. Female adult mice with intact ovaries were implanted subcutaneously with 17β-estradiol pellets (3.0mm, 60-day release, 0.36 mg/pellet; Innovative Research of America, FL, USA) 1 day prior to cell injection. Tumors were allowed to grow until palpable, at which time mice were injected with 0.5mCi of [ ^18^ F]FES into the tail vein and 50 minutes postinjection, imaged using a nanoScan whole body PET/MRI small animal scanner (Mediso, Budapest, Hungary). Scans were reconstructed using the Tera-TOMO software provided by Mediso.

## Results

[ ^18^ F]FES was synthesized in decay corrected yields of 67 ± 9% based on K[ ^18^ F]F (n = 34). Radiochemical purity was more than 96% and specific activity at the end of synthesis ranged from 75.2-134.8 GBq/μmol. The synthesis time including HPLC purification and reformulation was 93 minutes.

**Fig. 1:**
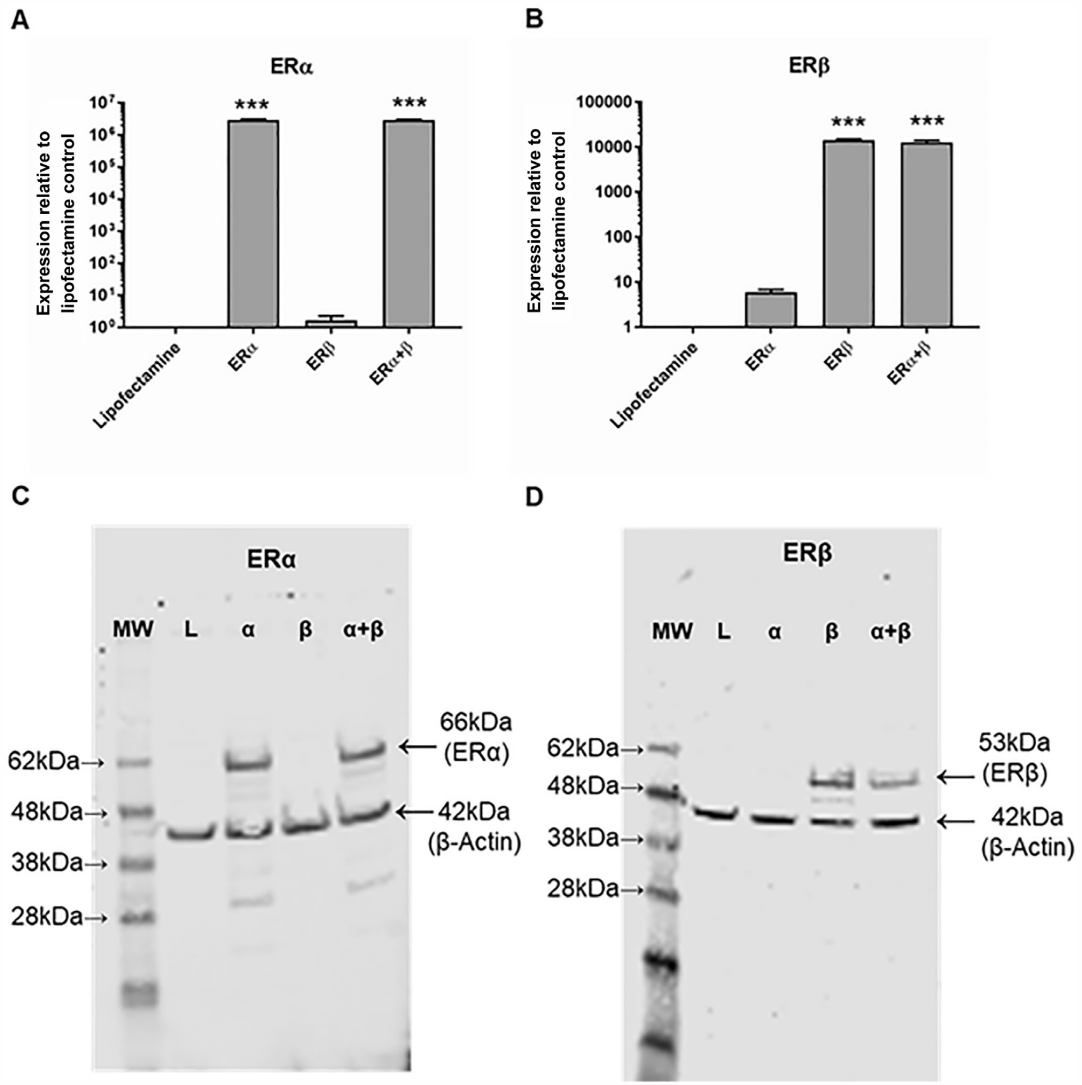
Transient transfection of HEK293T cells with ER subtypes. PCR ( **A** and **B** ) and Western blot ( **C** and **D** ) analysis of HEK293T cells either mock transfected (lipofectamine control), or transfected as indicated with either ERα, ERβ alone, or both. Panels show results for ERα PCR (A), ERβ PCR (B), ERα Western blot (C), and ERβ Western blot (D). Data in panels A and B show means ± SEM (n = 3) with significance of p < 0.001 (***) relative to lipofectamine control. ERα, estrogen receptor alpha; MW, molecular weight; L, lipofectamine; α, ERα-transfected; β, ERβ-transfected; α + β = transfected with both ERα and Erβ; PCR, polymerase chain reaction.

In order to characterize the binding properties of [ ^18^ F]FES, HEK293T cells were transfected with ERα and ERβ subunits, both alone and in combination. RT-PCR ( Fig. 1A, B ) and Western Blotting ( Fig. 1C, D ) analysis showed successful and specific mRNA and protein expression of expected ER subunits. ERα and ERβ subunits were not detected in untransfected HEK293T cells. Similarly, HEK293T cells transfected with only one subunit did not show expression of the alternate subunit.

**Fig. 2:**
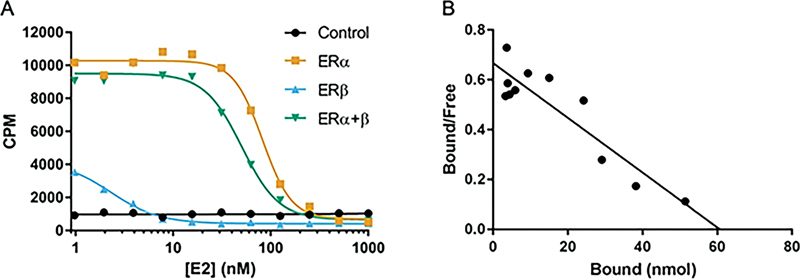
In vitro binding characteristics of [ ^18^ F]FES. Competitive binding ( **A** ) and Scatchard plot ( **B** ) of [ ^18^ F]FES binding to HEK293T cells transfected with ER subunits as indicated (A). Panel B was transfected with Erα alone. [ ^18^ F]FES bound to HEK293T cells transfected with Erα alone or Erα + Erβ but not Erβ alone (A). Scatchard analysis of Erα-alone transfectants showed a single high-affinity binding site of 94nM. ER, estrogen receptor; [ ^18^ F]FES, 16α-[ ^18^ F]fluoro-17β-estradiol.

Binding analysis revealed the [ ^18^ F]FES PET ligand bound only to the ERα subunit. In competitive binding studies, control and ERβ-transfected HEK293T cells did not show appreciable binding of [ ^18^ F]FES. HEK293T cells transfected with either ERα alone or with both ERα and ERβ bound [ ^18^ F]FES. Bound [ ^18^ F]FES was able to be competed off with cold estradiol ( Fig. 2A ), with an IC50 of 82nM (ERα alone) and 51nM (ERα and ERβ). The affinity of this binding was determined by Scatchard analysis to be 94nM for ERα alone and 51nM for ERα and ERβ combined ( Fig. 2B ). Given the lack of binding observed with [ ^18^ F]FES to ERβ, the biological activity of the transfected ERβ subunit was assessed by treatment of ERβ-transfected HEK293T cells with 1µM E2 for 24 hours. Real-time RT-PCR analysis demonstrating upregulation of GREB1, an ERβ-responsive gene, confirming the *ESR2* transfection construct used in these studies encoded biologically-active ERβ protein (see Supplementary Material Fig. 1 ).

**Fig. 3:**
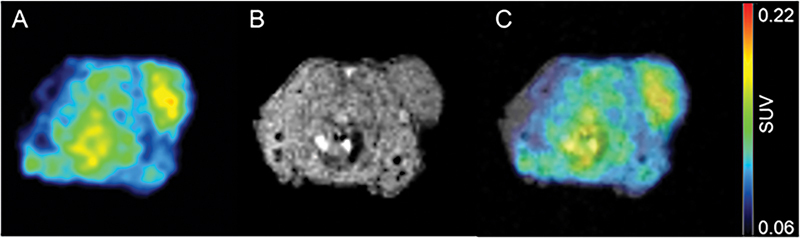
In vivo binding characteristics of [ ^18^ F]FES. Mice bearing MCF7 xenograft tumors showed uptake of the [ ^18^ F]FES PET tracer at the tumor graft site. Panels are an [ ^18^ F]FES PET transaxial image ( **A** ), magnetic resonance imaging (MRI) ( **B** ), and PET/MRI overlay ( **C** ). The color scale used indicates highest uptake of [ ^18^ F]FES in red and lowest uptake in black. [ ^18^ F]FES, 16α-[ ^18^ F]fluoro-17β-estradiol; PET/MRI, positron emission tomography/magnetic resonance imaging.

To determine whether [ ^18^ F]FES could bind to ERs in an in vivo setting, BALB/c nu/nu mice with MCF7 xenografts were injected with 0.3mCi of [ ^18^ F]FES. Mice were injected as soon as tumors became palpable, to minimize the presence of necrotic tissue and resultant nonspecific uptake with the tumor. Specific uptake of the [ ^18^ F]FES tracer was noted at the site of the tumor xenograft, as well as within the abdomen (see Fig. 3 for representative images).

## Discussion

This work has shown that [ ^18^ F]FES binds almost exclusively to ERα in vitro, with affinity comparable to native estradiol (18nM) 31 and with virtually no binding to ERβ. [ ^18^ F]FES is able to bind to ERα in vivo on MCF7 tissue grafts and be visualized in vivo. These results suggest that this tracer might be useful for identification of cancers predicted to have adverse biological characteristics due to ERα expression in vivo.

Slight variations in absolute binding affinity between [ ^18^ F]FES and ERs have been observed by various studies. 28 32 33 34 The fact that radiolabeled E2 binds with different affinities to cell lines in the same experiment 28 suggests that obtaining a precise and reproducible binding affinity is difficult and is likely dependent on the technical properties of the individual assay, the nature of the sample (e.g., purified receptor vs transfected cell line), and the unique biological properties of the cells/samples used.

The reason(s) why [ ^18^ F]FES has differential affinity for ERα and ERβ are not known. There is no reported crystal structure of [ ^18^ F]FES bound to ERs; however, there is anecdotal evidence that raises some possible mechanisms. First, the binding pocket of ERβ is reported to be smaller than that of ERα. 35 The presence of the additional fluorine atom may prevent the binding of [ ^18^ F]FES to ERβ by virtue of the fluorine atom being attached to the carbon atom at position 16, which sits within the deepest site of the receptor's ligand binding pocket. 36 Additionally, the highly hydrophobic fluorine atom is directly adjacent to the highly hydrophilic hydroxyl group attached to the carbon atom at position 17, potentially disrupting the charge-dependent interactions at this site. It is unlikely that the differential binding is caused by the amino acids making up the ligand binding pocket. The ligand binding pockets of ERα and ERβ differ by two amino acids; however, these changes are conservative and not in the region of the fluorinated carbon 16 atom. 36

ERs, understandably, have been most commonly studied in breast cancers, where they may be mutated, especially in the setting of recurrent disease. Studies have reported that [ ^18^ F]FES can bind to some (Y537S and Y537C) 33 but not all (no binding to G521R) 34 activating mutations of ERα in vitro. This would complicate the interpretation of [ ^18^ F]FES PET studies and may account for reductions in the positive predictive value of [ ^18^ F]FES PET, particularly in the setting of advanced breast cancer. 37 38

There is evidence that the de novo expression or constitutive activation of ERα can also play a significant role in cancers that are not commonly thought to be driven by estrogens, such as prostate cancer. The expression of ERα in prostate cancer might mediate adverse biological behavior of the cancer and hence its detection could be of clinical relevance as a prognostic or predictive biomarker. The known effects of ERα signaling in prostate cancer suggest that ERα-expressing prostate cancers might be more likely to metastasize or to exhibit more aggressive biological behavior than comparable ERα-negative cancers. ERα could be used in this setting as an escape pathway in the setting of androgen deprivation or AR inhibition. Patients with ERα-expressing prostate cancers might best first be directed to treatments such as cytotoxic chemotherapy or radionuclide therapy rather than AR-targeted therapies, especially if residual serum or tissue levels of estrogen are sufficient to activate ERα. Alternatively, such cancers might respond to specific blockade of ERα while maintaining AR inhibition, perhaps also in the context of activation of signaling through ERβ.

Agents now exist that can signal selectively through ERα and not ERβ, and vice versa. In breast cancer, tamoxifen has mixed agonist and antagonist effects; toremifene is an ERα antagonist; neither have significant clinical efficacy in prostate cancer. 39 40 41 Raloxifene is a relatively selective ERβ agonist 42 43 but again has limited if any activity in prostate cancer, either alone or in combination with an AR antagonist. 44 45 It is possible that effects on ERα may explain some of these discrepancies: many anti-estrogens including tamoxifen and raloxifene actually increase expression of ERα mRNA. 46 Newer SERMs, such as diarylpropionitrile and prinaberel (ERβ-selective ligands) or BHPI (HY-12825; ERα antagonist 47 ); and SERDs such as GDC-0810/ARN-810 or AZD9496 (promoters of ER degradation) are in development. Our study does not address these questions, which will require properly-designed clinical trials. In conclusion, our study describes [ ^18^ F]FES as a tool that might be suitable to identify patients whose tumor expresses ERα and could be selected for ERα-targeted therapies.
