# Supplementary material for: Characterization of an Estrogen Receptor α-Selective 18 F-Estradiol PET Tracer
Source: World J Nucl Med. 2024 Jun 18;23(3):153–60. doi: 10.1055/s-0044-1786518 (PMC11335392; doi:10.1055/s-0044-1786518)
Supplement: Supplementary file 1 — Supplementary Material [file 10-1055-s-0044-1786518-s2270004.pdf]

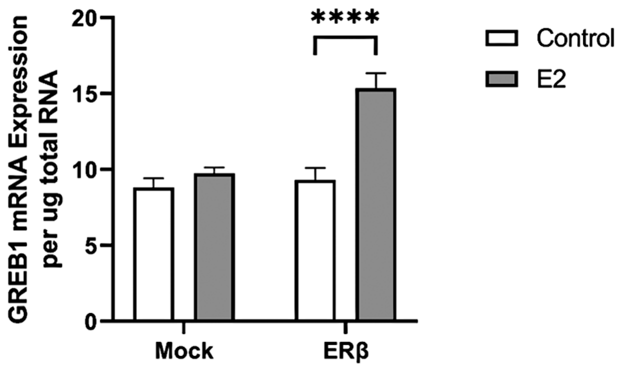

Supplementary material Fig. 1 Upregulation of GREB1 mRNA in ERβ-transfected HEK293T cells. PCR analysis of HEK293T cells either mock transfected (lipofectamine control) or transfected with ERβ alone and treated with estradiol as described in the Materials and Methods section. Data show means ± SEM (n = 3) with significance of $p < 0.0001$ (****). ERβ, estrogen receptor beta; PCR, polymerase chain reaction; SEM, standard error of mean.

 © 2024. The Author(s).
